# Proteome analysis of the prefrontal cortex and the application of machine learning models for the identification of potential biomarkers related to suicide

**DOI:** 10.3389/fpsyt.2024.1429953

**Published:** 2025-02-20

**Authors:** Manuel Alejandro Rojo-Romero, Nora Andrea Gutiérrez-Nájera, Carlos Sabás Cruz-Fuentes, Ana Luisa Romero-Pimentel, Roberto Mendoza-Morales, Fernando García-Dolores, Mirna Edith Morales-Marín, Xóchitl Castro-Martínez, Elier González-Sáenz, Jonatan Torres-Campuzano, Tania Medina-Sánchez, Karla Hernández-Fonseca, Humberto Nicolini-Sánchez, Luis Felipe Jiménez-García

**Affiliations:** ^1^ Programa de Doctorado en Ciencias Biomédicas, Universidad Nacional Autónoma de México (UNAM), Mexico City, Mexico; ^2^ Psychiatric and Neurodegenerative Diseases Laboratory, National Institute of Genomic Medicine, Mexico City, Mexico; ^3^ National Institute of Psychiatry “Ramón de la Fuente Muñíz”, Mexico City, Mexico; ^4^ Institute of Expert Services and Forensic Sciences of Mexico City (INCIFO), Mexico City, Mexico; ^5^ National Institute for Elderly, Mexico City, Mexico; ^6^ Cell Nanobiology Laboratory, Faculty of Sciences, National Autonomous University of Mexico, Mexico City, Mexico

**Keywords:** suicide, brain, dorsolateral prefrontal cortex, proteome, machine learning, potential biomarker

## Abstract

**Introduction:**

Suicide is a significant public health problem, with increased rates in low- and middle-income countries such as Mexico; therefore, suicide prevention is important. Suicide is a complex and multifactorial phenomenon in which biological and social factors are involved. Several studies on the biological mechanisms of suicide have analyzed the proteome of the dorsolateral prefrontal cortex (DLPFC) in people who have died by suicide. The aim of this work was to analyze the protein expression profile in the DLPFC of individuals who died by suicide in comparison to age-matched controls in order to gain information on the molecular basis in the brain of these individuals and the selection of potential biomarkers for the identification of individuals at risk of suicide. In addition, this information was analyzed using machine learning (ML) algorithms to propose a model for predicting suicide.

**Methods:**

Brain tissue (Brodmann area 9) was sampled from male cases (n=9) and age-matched controls (n=7). We analyzed the proteomic differences between the groups using two-dimensional polyacrylamide gel electrophoresis and mass spectrometry. Bioinformatics tools were used to clarify the biological relevance of the differentially expressed proteins. In addition, this information was analyzed using machine learning (ML) algorithms to propose a model for predicting suicide.

**Results:**

Twelve differentially expressed proteins were also identified (*t*_14_ ≤ 0.5). Using Western blotting, we validated the decrease in expression of peroxiredoxin 2 and alpha-internexin in the suicide cases. ML models were trained using densitometry data from the 2D gel images of each selected protein and the models could differentiate between both groups (control and suicide cases).

**Discussion:**

Our exploratory pathway analysis highlighted oxidative stress responses and neurodevelopmental pathways as key processes perturbed in the DLPFC of suicides. Regarding ML models, KNeighborsClassifier was the best predicting conditions. Here we show that these proteins of the DLPFC may help to identify brain processes associated with suicide and they could be validated as potential biomarkers of this outcome.

## Introduction

Suicide is the second leading cause of death among young people aged 15-29 years (1). Suicide has become a major public health problem worldwide, especially in countries where the majority of the population belongs to low- and middle-income countries, such as Mexico (2). The suicide rate in the country has steadily increased over the past four decades (3). Suicide is a complex and multifactorial phenomenon resulting from the interplay of biological, psychological and social correlates (4). Suicide is known to occur in the context of many psychiatric conditions such as major depressive disorder (MDD), and substance abuse (5). However, there is a lack of knowledge about the molecular mechanisms involved in suicide and no biomarkers have been stablished (6).

The neurotransmission system theory, the most studied theory on the neurobiological basis of suicide, describes impairments in this pathway, particularly in relation to the serotoninergic and dopaminergic systems. However, more information is becoming available every day, and current data from omics research suggests that other brain processes and molecules besides neurocommunication may be involved (7).

In fact, it has been reported structural changes and altered genome-wide DNA methylation and gene expression in the prefrontal cortex of individuals who died by suicide and their descendants (8, 9). These results suggest that the molecular composition is affected, as in the protein levels, whose role is closely related to structural changes in the brain. The dorsolateral prefrontal cortex (DLPFC) is of great interest in suicide research because it is involved in impulsivity, decision-making, emotional responses, and executive functions (10, 11). It is also part of the neurocircuitry associated with suicide (12). In addition to studies on the neurotransmitter system, some studies on the DLPFC of people who have died by suicide have found peculiarities in molecules from the endocannabinoid system (13) and hypothalamic-pituitary-axis (HPA) (9). In particular, structural and functional deficits reported in the DLPFC are associated with the severity of depression and suicidal behavior (8).

Proteins are essential for the function of the human brain as they regulate the homeostasis of mood, behavioral and cognitive processes (14). Alterations in the structure, function or expression of various proteins have been associated with various mental disorders, including suicide (15, 16). Proteomic analysis identifies differences in protein regulation under different physiological and environmental conditions (17, 18). In combination with bioinformatics, it is possible to identify and analyze the biologically enriched metabolic pathways in which candidate proteins are involved, making it possible to gain information about the molecular basis of complex phenotypes such as suicide (19). For this reason, we believe that proteomic analysis of post-mortem brain samples from suicide cases could provide direct insight into the molecular pathophysiology of this behavior to aid in the development of future predictive diagnostic tools.

In this context, previous studies have analyzed the protein content of prefrontal cortex or amygdala samples obtained from individuals who died by suicide (20–23), identifying distinct proteomic profiles compared to control individuals.

To our knowledge, there are no reports on the cerebral proteome of Latin American suicide cases. Using a mass spectrometric untargeted proteomics approach, this study aimed to identify differentially expressed protein patterns in the DLPFC of suicide cases compared to age-matched controls among Mexican nationals. We also attempted to integrate the identified molecules into interaction pathways with a biological network. Since the proteome is highly dynamic and influenced by internal and environmental stimuli, we hypothesized that the protein profile of suicide cases will differ from that of the control group.

In addition, the use of artificial intelligence (AI) to study and predict risks to physical and mental health is an emerging and exciting area of research that can be leveraged with data from new generation technologies (NGTs) such as proteomics. The study by the group of Simon and cols applied ML techniques to analyze electronic health records of individuals who had outpatient visits (24). The researchers developed a model that predicted the risk of suicide attempts and suicide deaths based on various factors such as psychiatric diagnoses, medical history, and recent health care use. One study used ML algorithms to predict suicide phenotypes based on gene expression in blood (25). Kessler and cols used ML methods to examine heterogeneity in response to treatment for MDD (26). By analyzing self-report data, the researchers developed a predictive model that identified subgroups of individuals with different treatment responses, including those at higher risk for suicidal ideation.

The present study sought to identify differentially expressed proteins and active biochemical signaling pathways in the DLPFC of Mexican suicide cases. This knowledge was applied to strengthen the use of protein biomarkers that could be monitored in suicidal individuals, but also to test a machine learning model to predict which samples are from individuals who have died by suicide. Furthermore, these would be potential biomarkers of suicide risk in individuals if they could be monitored by peripheral samples. Therefore, we trained a machine learning (ML) model with densitometric data from our proteomic analysis to investigate whether this data is sufficient to detect whether a sample is from a suicide case or a control subject.

## Methods

### Brain tissue collection

This study was conducted in accordance with the ethical principles of the last Declaration of Helsinki; with ethical approval was granted by the National Institute of Genomic Medicine (INMEGEN: CEI#2016/33) and the Institute of Forensic Sciences of Mexico City (INCIFO: CEI#015/2022). To ensure confidentiality, a non-repeatable and non-identifiable code was assigned to each suicide case and control.

Postmortem tissue from the DLPFC (Brodmann area 9) was taken from the deceased within 24 hours as part of the legal autopsy. A tissue block of approximately 5 cm^3^ from the left hemisphere was sectioned, rapidly frozen without fixation and transported on dry ice to INMEGEN where it was stored at -80°C until further use.

The subjects’ records were collected with information such as demographic data, death certificates, autopsy reports, toxicology reports, substance abuse, suicidal behavior, suicide notes (if available), and statements from relatives or witnesses of death. Drug abuse was identified in the toxicology reports: cocaine, heroin, amphetamines, opioids; or suspected intoxication, such as rodenticides. Ethanol, methanol or various types of medication were also measured.

In Mexico, and in accordance with National Code of Criminal Procedures, a coroner must identify the causes of all uncertain or violent deaths, including all potential cases of suicide. Therefore, each possible suicide is subject to an investigation conducted by forensic pathologists and police officers, providing data to enable the coroner’s office to generate a formal verdict regarding manner of death. The records from the coroner involving suicides generally include demographic information, circumstances of the death (as reported by witness reports), acute, and chronic stressful life situations, autopsy and toxicology reports, police investigation records, medical and psychiatric reports from hospitals, suicide notes, and insurance data. In Mexico City, the Forensic Science Institute (INCIFO) is the institution that garners all suicide cases for the inhabitants of the city.

According to the criteria of the Statistical Manual of Mental Disorders, Fifth Edition (DSM-5) and with the information obtained from the coroner’s record, a consensus diagnosis conducted by a pathologist, a specialist in psychological autopsy, and a psychiatrist was reached for each suicide case.

### Tissue lysis

The DLPFC were weighted (0.5 grams) and immersed in 1.5 mL of a cooled buffer containing a cocktail of protease inhibitor (Cat. 05892791001 Roche), 7M Urea, 2M Thiourea, 30 mM (Tris-[Hydroxymethyl]-aminomethane), and 4% CHAPS (3-[(3-cholamidopropyl)dimethylammonio]propane-1-sulfonate) with a final pH of 8.0 (26). Samples were homogenized manually using plastic pistils (Cat. 26351Bel Art, CTR Scientific) until pieces of the tissue were no longer visible. Lysis was completed with sonication at 60 Hz during 15 sec, three times, with 1-minute pauses between each interval. Then samples were centrifuged at 12,000 g for 15 minutes at 4°C and the aqueous phase containing proteins was collected.

### Protein extraction

The protein extraction was performed (27). In brief, a total of 300 μL of lysate was mixed with 900 μL of methanol and 300 μL of dichloromethane and centrifuged at 12,000 × g for 20 min at 4°C. The aqueous phase containing cell debris was discarded, while the solid phase containing the proteins was resuspended in 900 μL ethanol and centrifuged again for 2 minutes. The supernatant was discarded and the pellet was dried at room temperature. The extracted proteins were resuspended in 1.5 mL TRIS-HCl (50 mM, pH 7.4) and stored at -80°C. Protein concentration was determined using the Lowry-Peterson method with bovine serum albumin (BSA) as standard (28). Protein integrity was tested by one-dimensional electrophoresis (29) (**Supplementary Figure S1**, **Supplementary Table S1**).

### 2D gel electrophoresis

Protein integrity was evaluated by denaturing one-dimensional gel electrophoresis with 20 μg total protein per lane on a polyacrylamide gel (12%). Samples were then focused for 2D isoelectrofocusing (IEF) on 7 cm long IPG strips with a pH gradient of 4-7 (Cat. 17-6001-10 GE Healthcare Amersham Biosciences) using 100 μg total protein in 125 μl rehydration buffer (8M urea, 4% w/v CHAPS, 40 mM DTT and 2% v/v IPG; Amersham Biosciences) for 10 h. Each strip was initially focused at 7100 V/h, followed by progressively increasing voltage. After focusing, the strips were immersed in equilibration buffer (6M urea, 50 mM Tris-HCl, 30% v/v glycerol, 2% w/v SDS, 0.002% w/v bromophenol blue) and 100 mM DTT for 15 min, and then 250 mg iodoacetamide was added. The strips were shaken for 15 minutes. The second dimension (SDS-PAGE) was performed on a 12% polyacrylamide gel at 60 V for 20 min, and the voltage was gradually increased to 120 V. The geles were stained with Sypro Ruby (Cat. S12000 Thermo Fisher Scientific) following the manufacturer’s instructions. Gels were visualized using a Universal Hood III (ChemiDoc MP Imaging System, Bio-Rad). Master gels displaying a complete overview of protein expression within a 4-7 pH range in suicide cases and paired controls were obtained.

### Image analysis

The densitometric analysis and data normalization (by quantiles) were achieved with PDQuest (Version 8.0.1; BioRad,CA) software. The automatic spot detection was performed following the recommended parameters, which included the minimal area, smoothing factor, and larger cluster. Digital master images were created for easier comparison between the control and suicide samples, thus establishing the average protein profile of each group and using the densitometric values obtained in the statistical analysis. The wizard detection method proposed by the BioRad PDQuest software, was used to detect the spots. The gels were automatically matched in order to attribute a common spot identity for the same spots derived from different images. Manual editing was carried out. For each sample, when a protein was detected in all the replicates, this protein was automatically added to the master gel.

### Statistical analysis

Statistical analysis was done with the MetaboAnalyst software v4.0 (30). A Mann-Whitney U test was applied to compare the postmortem interval in both conditions. In contrast, the Student’s *t*-test (t_14_ or t_w14_ when W_(1,14)_ ≥ 0.05) was used to compare the variable of age and the densitometric values of each protein in cases and controls. The fold change (FC) values were estimated with the following formula: [log2 (S/C)], where S corresponds to the suicide value, C is the control value, and log2 represents the base 2 logarithm. All protein spots identified as significantly different (FC ± 0.30 and P ≤ 0.05) were selected for MS analysis. The Cohen’s *d* test for the size effect of each pair comparisons was applied. The top six proteins up or down-regulated in suicide cases were selected.

### Protein identification

According to the statistical analysis the differentially expressed proteins (FC ± 0.30 and P ≤ 0.05) were manually extracted from the gel. These gel fragments were dehydrated, faded with acetonitrile (ACN):50 mM v/v, ammonium bicarbonate (NH4HCO3) (1:1), and digested with trypsin (Promega V528A) for 18 h at 37°C. The released peptides were concentrated using a vacuum concentrator (Eppendorf 5301) and desalted using a Zip TipC18 (Cat. Z720070 Millipore). Samples were loaded into an α-cyano-4-hydroxycinnamic acid matrix and analyzed using a MALDI-TOF/TOF 4800 analyzer. The mass spectrometry data were matched against the Paragon database (ProteinPilot) to identify candidate proteins with at least 66% reliability. The densitometric and fold change values, just as the identity of the proteins, were uploaded to MetaboanalystR software v4.0 (30) to create a heatmap, and a Principal Component Analysis (PCA) was used to improve the visual representation of the results.

### Biological pathway analysis

The Uniprot access code of the identified proteins was used as an input in MetaCore (Clarivate Analytics) and String (ELIXIR) to determine the activation/inhibition and the interaction of the proteins through a biological network. Additionally, a list of cellular pathways was obtained from String software. Those with an FDR value above 0.1 were discarded to obtain the most enriched pathways.

### Western blot

To test if the differences identified in the 2D/MALDI TOF were replicable in a larger cohort, a pair of the underexpressed proteins, Alpha Internexin [INA (G-9)] and peroxiredoxin 2 [PRDX2 (A-2)], were quantified by western blot using a different set of samples (31). In summary, the DLPFC was homogenized with 1 ml of RIPA buffer with protease inhibitor (Complete, Roche, cat.: 11697498001), centrifuged at 15,000 rpm for 30 minutes, and the supernatant was obtained. Protein concentration of the supernatant was determined using micro-BCA Protein Assay Kit (ThermoFisher, Cat. 23235) according to the method indicated by the supplier. Absorbance measurement was performed at 562 nm in a plate reader (Epoch, Biotek). Aliquots of protein samples (30 μg) were separated by electrophoresis on a SDS -polyacrylamide gel. The separated proteins were then transferred to PVDF membranes. Membranes were blocked with 5% bovine serum albumin (BSA) or 5% nonfat milk in TBS and Tween-20 at room temperature for 1 hour. The membranes were then tested overnight at 4°C with primary antibodies against the proteins Alpha Internexin (INA (G-9), Cat. SC-271302) and PRDX2 (Cat. SC-515428) (1:1000), and β-actin (1:5000) as loading control. All of these antibodies were supplied by Santa Cruz Biotechnology. After three brief washes in TBST, the membranes were incubated with an HRP-conjugated secondary antibody (Abcam; ab6759). Immune complexes were detected by chemiluminescence (Western Bright ECL HRP substrate, Advansta). Immunoblotting was scanned and quantified using Molecular Image. ChemiDoc™ XRS+ with Image Lab™ software.

### Classification analysis using machine-learning algorithms

For classification analysis, several libraries in Python NumPy a powerful numerical computing library, Pandas a open-source data manipulation and analysis library, and from Scikit-learn, the StandardScaler class (from sklearn. preprocessing module) for standardizing features by removing the mean and scaling to unit variance, and Column Transformer (from sklearn. compose module) were used. The library matplotlib. Pyplot was used for plotting. Module sklearn.model_selection provided a flexible set of tools for data-splitting model evaluation and allowed the building and assessment of ML models effectively. We used the train_test_split function of this module, and the ML models GaussianNB, KNeighborsClassifier, Perceptron, and MLPClassifier to train the datasets. We obtained the metrics using tools from the sklearn.metrics module. This module provides evaluation metrics to assess the performance of machine learning models, including accuracy, precision, recall, F1-score, ROC curve, and confusion matrix. These metrics can be used to measure the quality of predictions and compare different models. We used an accuracy and confusion matrix to evaluate the models using data frames. We used the X matrix, protein data, and y vector (out) for the control (no suicide) and suicide conditions. These data frames were divided into X_train, y_train, X_test, and y_test to test the ML models. The optimization process was performed with the Adam optimizer, and the optimized parameters were as follows: best test loss= 0.12563762068748474, batch size= 4, and learning rate= 0.001. The tested models performed well on both the training and test datasets.

## Results

### Sample characterization

Suicide completers died by self-injury, as determined by the coroner. No self-inflicted injuries or pre-existing medical conditions were identified in controls. Complete data regarding the coroner´s records are described in a previous article (3). Only the cases with enough information to reach the consensus diagnosis were used in this study.

The examined brain tissues belonged to male suicide cases (n=9) and age-matched controls (n=7). **Table 1** shows the sociodemographic information of the samples. Regarding age and PMI, there were no statistical differences among controls and cases (*t*= 0.51; *p*=0.61 and U=22; *p*=0.19, respectively). Concerning suicide, all cases were men who died by hanging, and all of the controls died by gunshot wounds. Most of the cases presented a psychiatric diagnosis for MDD (88%) and just a few for alcohol use disorder (12%). Controls were free from previous psychiatric diagnoses. No individual from the total cohort tested positive for substance abuse at the time of death, but 88% of the suicide cases tested positive for alcohol. At the same time, controls did not present this feature.

**Table 1:** Characteristics of sudden death controls and suicide cases.

Variable	Sudden death controls	Suicide cases	Statistical analysis
**Gender**	Male (100%)	Male (100%)	**-**
**Age, mean** ± **SD**	30.71 ± 1.44	30 ± 1.95	*t*_(14)_= 0.51, *p*=0.61
***Postmortem* interval (PMI) in hours**	12.2 ± 0.78	14.2 ± 2.76	*U* = 22, *p*=0.19
**Cause of death**	Gunshot wound (100%)	Hanging (100%)	–
**Psychiatric disorder (DSM-V diagnosis)**	None	MDD (88%) AUD (12%)	–
**Alcohol use at the time of death**	None	88%	
**Substance use at the time of death**	None	None	

SD, Standard deviation; *t*, Student’s t-test; *U*, Mann-Whitney U-test; *p*, p-value; MDD, Major depressive disorder; AUD, Alcohol use disorder. Bold Values are significant at p <0.05.

### 2D-PAGE protein profiling and protein identification by mass spectrometry

**Figure 1:**
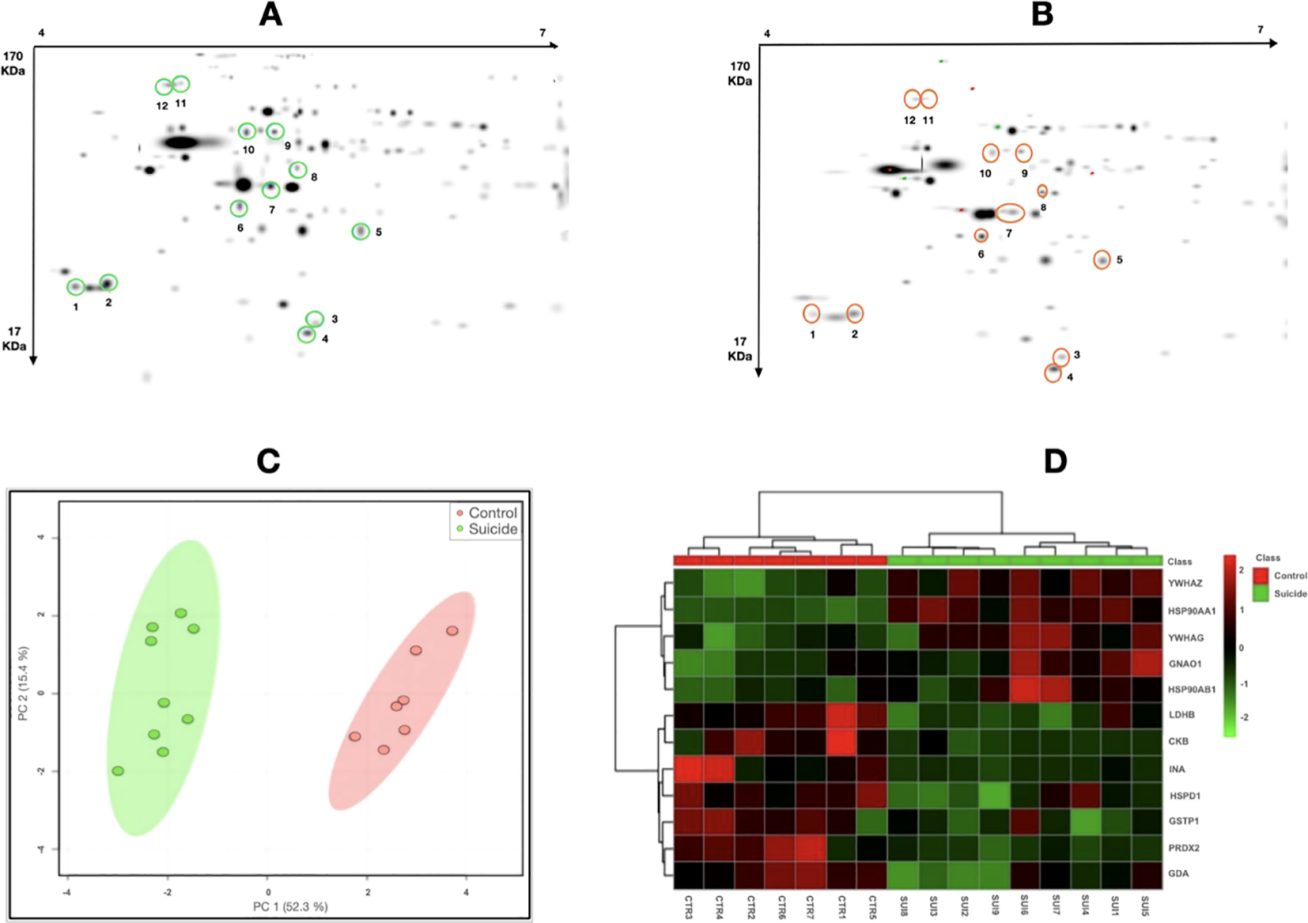
2D-PAGE protein profile. Master gels of **(A)** protein profile of suicide cases and **(B)** protein profile of controls with sudden- death controls. Differentially expressed proteins are marked in circles. Cluster analysis. **(C)** Principal Component Analysis score plot for the combined data. PC1 and PC2 account for 67.7% of the total variation. Suicide cases are shown in green and the sudden- death controls in red. **(D)** Heatmap of protein expression. The green (suicide cases) and red (controls) bars at the top of the graph illustrate the physiological state of the individuals. The light red indicates upregulation, while the deep green indicates downregulation; black indicates no change in expression.

A pattern of the master gels was obtained from the two-dimensional electrophoresis, followed by PDQuest analysis, and the selection of six down- and six up-regulated proteins in suicide cases compared to controls (illustrated in **Figures 1A, B**).

To identify these differentially expressed proteins, it was used high-accuracy LC-MS/MS, and the obtained results were matched against the ProteinPilot database (66% confidence). The results are shown in **Table 2**.

**Table 2:** Differentially expressed proteins in suicide´s DLPFC.

Protein Name	UniProt access code	GO Biological process	Log2 FC	Statistical analysis	Change in expression
Creatine Kinase B type	CKB	Phosphocreatine biosynthetic process, development of the substantia nigra (22, 23)	-1.01	*t*w(6.65)=3.55, *p*=0.01 W(1,14)=4.69, *p*=0.05 *d*=2.013	Downregulated
L-Lactate Dehydrogenase Chain B	LDHB	Pyruvate metabolism (24)	-0.95	*t*_(14)_=5.83, *p*=0.00004 W(1,14)=2.3, *p*=0.15 *d*=2.9	Downregulated
Alpha - Internexin	INA	Neurofilament cytoskeleton organization, development of the substantia nigra (23, 25)	-0.90	*t*w(6.2)=3.058, *p*=0.02 W(1,14)=8.97, *p*=0.01 *d*=1.74	Downregulated
14-3-3- Zeta/Delta Protein	YWHAZ	Synaptic maturation, cytoskeleton rearrangement (26)	-0.49	*t*_(14)_=-5.07, *p*=0.0002 W(1,14)=0.15, *p*=0.7 *d*=-2.54	Downregulated
Peroxiredoxin 2	PRDX2	Oxidative stress response, apoptosis (27, 28)	-0.49	*tw*(6.56)=4.194, *p*=0.005 W(1,14)=7.45, *p*=0.016 *d*=2.38	Downregulated
Glutathione S- Transferase Pi	GSTP1	Oxidative stress response, neurodevelopment (25)	-0.36	*t*_(14)_=2.80, *p*=0.014 W(1,14)=0.15, *p*=0.7 *d*=1.41	Downregulated
Heat Shock Protein 90-beta	HSP90AB1	Axon extension, central nervous system neuron axogenesis, Unfolded proteins response (25, 29)	1.75	*t*_(14)_=2.74, *p*=0.016 W(1,14)=1.52, *p*=0.237 *d*=-1.38	Upregulated
GNAO1 Protein	GNAO1	G Protein activity, dopamine receptor signaling pathway (26)	1.03	*t*_(14)_=-2.67, *p*=0.018 W(1,14)=1.83, *p*=0.2 *d*=-1.35	Upregulated
Guanine Deaminase	GDA	Guanine metabolism, nervous system development (22, 30)	1.03	*t*_(14)_=3.64, *p*=0.003 W(1,14)=2.14, *p*=0.17 *d*=1.83	Upregulated
Heat Shock Protein 90AA1	HSP90AA1	Axon extension, central nervous system neuron axogenesis, Unfolded proteins response (25)	1.027	*t*w(9.37)=-9.58, *p*=0.00002 W(1,14)=4.59, *p*=0.05 *d*=-4.3	Upregulated
14-3-3 Gamma Protein	YWHAG	Neuron differentiation and synaptic plasticity (31)	0.53	*t*_(14)_=-3.42, *p*=0.004 W(1,14)=0.98, *p*=0.34 *d*=-1.72	Upregulated
Heat Shock Protein 60 KDa	HSPD1	Unfolded proteins response (22, 32)	0.52	*t*_(14)_=3.21, *p*=0.006 W(1,14)=0.94, *p*=0.34 *d*=1.61	Upregulated

*t*, Student’s t-test; *tw*, Welch’s t-test; W, Levene’s test; *d*, Cohen’s *d* test; *p*, p-value, degree freedom for the test is in subscript.

A Principal Component Analysis (PCA) was determined and the PC1 and PC2 scores were obtained from these 12 protein spots accounted for 67.7% of the total variance, thus allowing a graphical separation of the two groups in the heatmap (**Figures 1C, D**).

### Bioinformatics analysis

**Figure 2:**
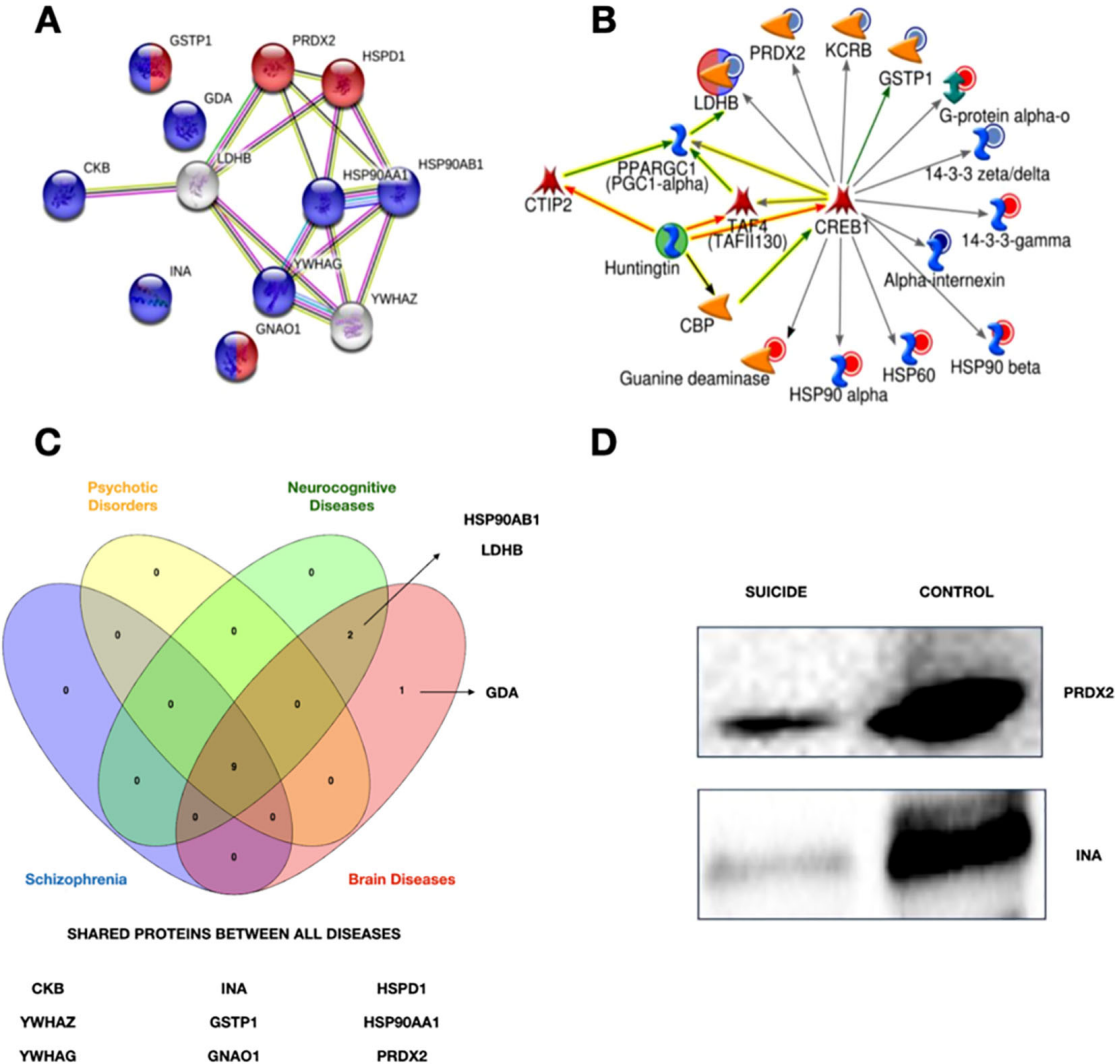
**(A)** Network obtained using string. Purple circles show the differentially expressed proteins related to neurodevelopment, while the red ones are involved in the oxidative stress response. **(B)** Network obtained through MetaCore. The differentially expressed proteins are linked to the transcriptional factor CREB1. Circles surrounding proteins show the changes in suicide cases. Blue circles represent a low expression of these proteins, and red ones mean an upper expression for controls. **(C)** Venn Diagram of associated diseases. Four of the ten associated diseases with the identified proteins. Created with Venny 2.0 with the information of **Supplementary Table S3**. **(D)** Western Blot. Validation of the down-expression of PRDX2 and INA in suicide cases.

The putative biological relevance of the differentially expressed proteins was addressed through network analysis. Using the UniProt access code in the STRING protein database, hypothetically enriched networks that included the previously identified candidate proteins were created. Two pathways were selected based on the criteria of the highest number of candidate proteins participating in the network and the FDR standards (**Figure 2A**).

A network of possible interactions (activation/inhibition) between the candidate proteins and other relevant molecules, of which we highlight CREB1, was generated using MetaCore software (Fig.2B). With the same software, **Supplementary Table S3** was generated with information about diseases related to the candidate proteins. **Figure 2C** summarizes the proteins shared between four of the ten associated diseases. A WB analysis was performed to validate the proteomic differences discovered by LC-MS/MS and bioinformatic analysis. As shown in **Figure 2D**, this analysis confirmed the significantly modified expression of the candidate proteins, including Alpha-Internexin (INA), and Peroxiredoxin 2 (PRDX2).

### Analysis with machine learning algorithms

The dataset included the protein levels obtained from the images of the 2D gels. The ML models GaussianNB, KNeighborsClassifier, and Perceptron were trained using the dataset. The dataset was split into a 70% and a 30% of the data for the training and test datasets, respectively. The aim of training this method was to correctly classify individuals according to their diagnosis [suicide (1) and no suicide (2)]. These three models fit the data (accuracy = 1). Individuals [three with no suicides (1) and one with suicide (2)] were tested for prediction and adequately predicted. These types of experiments based on proteomics and other omics sciences are sciences that, although with a limited number of samples, have the great advantage of collecting a large amount of information from the sample. To prevent overfitting and ensure that the model performs well on data outside the training set, cross-validation was performed, which is a statistical technique used in machine learning to assess how well a model generalizes to unseen data. The mean accuracies obtained for each model were 0.833, 0.93, and 1 for the Perceptron, GaussianNB, and KNeighborsClassifier models, respectively. The best performance was achieved by the KNeighborsClassifier. Thus, the levels of the 12 proteins identified by proteomics are potential biomarkers for classifying individuals at risk of suicide.

## Discussion

The results of genetic, genomic and imaging studies support the neurobiological basis for suicidal behavior. However, many of these studies have been conducted with peripheral samples (e.g. blood) from living individuals at high risk of suicide, who may differ in their clinical and molecular characteristics from those who have killed themselves. The major limitation is the access to the target organ associated with suicide: the brain. Current technologies for studying human brain samples from living individuals are limited to imaging and biopsies, but these are invasive and may pose several risks. The use of postmortem brain samples therefore allows us to further investigate suicide using methods to measure parameters that may be involved in the pathophysiology of this outcome, such as proteins and their regulatory processes.

The identification of altered patterns in the composition of the brain proteome of individuals who have died by suicide may provide important insights into the putative molecular mechanisms. In this study, a prototype proteomics pipeline of 2D gel electrophoresis and LC-MS/MS was applied to postmortem DLPFC samples. The result was the identification of 12 proteins that exceeded the statistical comparison cut-off in suicides compared to non-suicides. Half of them were downregulated, while the other half showed upregulated expression. The *in silico* analysis performed with String mapped them to two important biological processes: oxidative stress response and neurodevelopment (**Figure 2A**).

The creation of an activation pathway with all differentially expressed proteins was made possible with MetaCore software (**Figure 2B**). It is noteworthy that the analysis identified CREB1 as a molecular hub that has the ability to modulate the expression of the candidate proteins identified in this study. From the proteomic profile, it can be inferred that CREB1 is able to both up- and down-regulate these proteins, but the exact underlying mechanisms are still unknown. Although our analysis was unsupervised, there is a possibility that our results correlate with several diseases associated with suicide, such as schizophrenia and psychotic disorders (**Supplementary Table S2**; **Figure 2C**).

Only a few similar studies have been conducted so far, and **Table 3** summarizes the main results of these studies.

**Table 3:** Proteomic studies in suicide research.

Authors (year) Nation	Sample	Gender (Male/Female)	Age mean ± SD	Comorbidities in suicide cases	Differentially expressed proteins (DEPs)	Biological process/enriched pathway
Schilts et al. (2007) Germany	PFC S= 17 C= 9	S: 10/7 C: 7/2	Msu: 31.7 ± 11.17 Mc: 30.5 ± 11.6	MDD: 4 SBH: 2 AUD 3 BP: 1 CPS:1	GFAP SOD2 CRYAB	Cytoskeleton dynamics Oxidative stress response Neurotransmission Neurodegeneration
Kékesi et al. (2012) Hungary	PFC and Am S= 6 C= 6	Only males	S: 52.7 ± 14.2 C: 64.8 ± 17.2	NA	CBR1, DPYSL2, EFHD2, FKBP4, GFAP, GLUL, HSPA8, NEFL, NEFM, PGAM1, PRDX6, GPX1, SELENBP1, VIM, ACTB, CTSD, GFAP, TUBA2, **CKB**, **INA, YWHAG, YWHAZ**	Metabolism REDOX system/Oxidative stress response Cytoskeleton dynamics Synaptic function
Cabello et al. (2020) U.S.A.	DLPFC S= 5 C= 5	Only males	S: 52 C: 56	BPD1: 4 MDD: 1	KCNQ3, MBLAC1, TRIM36 RBMX, ADCY5, ENTPD2, NEK7, SNX5, FAH, MLC1, MBNL1, CBPC1, HCN2, PLCL1, DOCK1, CNTN4, CNKSR2, CAMK2D, EIF4E, UBE4A, SRR, GABRB1, MPP2, EARS2, EWSR1, SNX14, ARAP1, MAOA, ARHGEF9, PYCARD, ASMTL, GOLT1B, C9orf72	GABA, serotonin and dopamine receptor signaling Melatonin signaling CREB signaling in Neurons
Kim et al. (2021) South Korea	DLPFC S: 23 C: 13	S: 11/12 C: 13/0	S: 48.8 ± 13.6 C: 51.5 ± 12.9	SZ: 7 MDD: 4 None: 12	GSTT1, CAPNS1, CDC42, PTP4A2, CD46, CSNK2B, **CREB1**, ERF, NF-KB, ABRACL, UBE2P3, COL4A1, CD82, RPS4Y1, S100A10, MRGPRFG	Endocannabinoid pathway Apoptotic pathway Central nervous system development
Rojo et al. Current results (2024) Mexico	DLPFC S: 9 C: 7	Only males	S: 30 ± 1.95 C: 30.71 ± 1.44	MDD: 8 AUD: 1	CKB, LDHB, INA, YWHAZ, PRDX2, GSTP1, HSP90AB1, GNAO1, GDA, HSP90AA1, YWHAG, HSPD1	Nervous system development Oxidative stress response

Although the neurological circuitry associated with suicide is complex and involves multiple brain regions, the prefrontal cortex, particularly the DLPFC, has emerged as the most studied area in relation to the proteome of suicide. This may be attributed to the crucial role of the prefrontal cortex in decision-making and cognitive processes, impairments of which are associated with suicide and suicidal behavior (32). All four studies involved male subjects and only two involved female samples. According to the suicide paradox, the ratio of suicide in men to women is 4:1, although suicide attempts are more common in women. It is worth noting that this fact could facilitate the procurement of postmortem samples from males. Nevertheless, a comprehensive understanding of suicide requires an equal study of both sexes. Furthermore, it is important to recognize that the underlying cause of this ratio remains unknown, and whether it is congenital or genetic has yet to be determined (33). Therefore, it is imperative to examine the specifics of suicide in men and women separately before comparing the results to gain a more comprehensive understanding of gender differences in suicide.

Our study found that there were some similarities with Kékesi’s results (21), particularly in the change in down-expression of CKB in suicide cases. However, we observed opposite effects in INA, YWHAG and YWHAZ, namely down-expression in our samples compared to up-expression in their analysis.

It is worth important that certain groups of proteins, such as the peroxiredoxins (PRDX), the glutathione S-transferases (GST) and the families of heat shock proteins (HSP), are present in our analysis, even though they are not exactly the same. What is particularly exciting is that standard affected biological processes were discovered, even though the studies were conducted in four different populations, using different techniques and given the intrinsic heterogeneity of suicide phenotypes. The responses to oxidative stressand neurodevelopment are discussed in more detail below.

Given the exceptionally high energy demands of the brain, large amounts of reactive oxygen species (ROS) are released from the metabolism and the electron transport chain in the mitochondria (34, 35). Therefore, an efficient biological response to oxidative stress is essential for normal brain function.

Increased oxidative stress has been described in various mental disorders in which suicidal behavior is not uncommon (e.g. anxiety, schizophrenia, bipolar disorder). However, its presumed causal role appears to be far from clear. In a recent study, the concentrations of various biomarkers for oxidative stress were measured in the blood of people with self-injurious behavior and suicidal thoughts and attempts with various concomitant psychiatric illnesses. The authors point out that the response to oxidative stress varies across the spectrum of psychiatric diagnoses and suicidal behavior. However, they conclude that this process plays an essential role in the pathophysiology of suicide (36).

Some of the proteins identified here play an essential role in the chemical homeostasis of the human brain and the response to oxidative stress:

GSTP1 plays a crucial role in the glutathione metabolic pathway and is the most highly expressed isoenzyme of the GST family in the cerebral cortex (37). It helps to catalyse electrophilic compounds and the production of water-soluble products, which is highly dependent on glutathione (GSH), an important antioxidant (38). In addition, members of the GST family are also involved in various other processes, including cell proliferation and cell death (39). Low levels of GSTP1 have been linked to various neurodegenerative diseases such as Parkinson’s and Alzheimer’s (39, 40), neurodevelopmental disorders such as autism spectrum disorders (41) and mental illnesses such as schizophrenia and bipolar disorder (39, 42). Previous studies have also linked other members of the glutathione pathway to suicide. For example, GSTT1 was identified by Kim and colleagues, while Kékesi’s study found GSTM3 and GPX1 as differentially expressed proteins in the cases. The results of three of the five proteomic studies on suicide to date suggest that altered molecules in glutathione metabolism may be a characteristic process in the suicidal brain. However, further studies are needed to confirm these findings.

GNAO1 belongs to the family of G proteins. The seventeen known members of this family have been categorized into inhibitory G proteins (Gi), stimulatory G proteins (Gs), G9/11 proteins, G12/13 proteins and “other” G proteins (Go) (43). Go proteins are expressed throughout the nervous system (44). The protein mediates serotonergic, dopaminergic, GABAergic and cholinergic signaling pathways as well as the response to opioids and cannabinoids (45). In addition, data suggest that Go proteins are active mediators of neuronal calcium channel activity, as they can inhibit action potential in synapses (44). Interestingly, GNAO1 is associated with schizophrenia (46) and depression (47).

PRDX2 is an antioxidant enzyme that reduces hydrogen peroxide and can generate free hydroxyl groups. Hydrogen peroxide may also play a positive role in the brain as it is involved in cell differentiation, proliferation and migration (48). PRDX2 is frequently released under inflammatory conditions and its low expression is associated with oxidative stress and apoptosis (49). The enzyme has been associated with bipolar disorder (50) and schizophrenia (51). In a proteomic analysis of cerebrospinal fluid (CSF), PRDX2 was found to be less expressed in suicide cases. The authors hypothesize an impairment of the oxidative stress response in suicide cases caused by low levels of this critical enzyme (52). We also found low levels of PRDX2 in the suicide group. To validate our results, we performed a Western blot analysis with an independent study cohort of cases and controls that had similar characteristics to the samples used in the proteomic analysis. **Figure 2D** shows a tendency for decreased expression of PRDX2 in our samples. In addition, PRDX6 was also found to be a down-expressed protein in the analysis of Kékesi. The results of three independent studies indicate a general impairment of the PRDX family in suicide cases, highlighting these antioxidants as an important group of molecules in suicide research.

The development of the central nervous system is an extensive process controlled by many proteins that regulate several specific processes. These proteins are required for balance during neurodevelopment. However, changes in expression can also promote an inflammatory response with severe consequences for the developing and adult brain (53). In particular, abnormal neuronal development leads to defective connectivity of the nervous system, which affects brain circuitry and results in non-adaptive behavior (54) - impairments that have been associated with suicidal mental disorders. The role of the identified proteins associated with neurodevelopment is discussed below.

CKB and LDHB are both important proteins involved in bioenergetics. If there is an energy deficit, this can lead to cell death under stress conditions (54). CKB plays a crucial role in the conversion of ADP to ATP through the phosphorylate-creatine B cycle. It can also store energy (CKBP), which regulates the regeneration of ATP. Interestingly, a low expression level of CKB was found in postmortem brain samples from people with schizophrenia (55). While our analysis and previous reports suggest that the downregulation of this protein is significant, it is important to confirm these results by Western blot or other validation methods in a larger cohort. The deficit of this enzyme could indicate low energy activity in the brain of suicide cases. On the other hand, LDHB is a glycolytic enzyme that converts lactate, an important energy substrate in the brain, into pyruvate. Deficiency of LDHB has been associated with oxidative stress and neuroinflammation/neurodegeneration in adult mice through knock-out studies of this gene (56). It has also been associated with MDD and suicidal behavior (57, 58).

YWHAZ and YWHAG belong to the family of 14-3-3 cross-link proteins that are highly conserved in eukaryotic cells. These proteins have a wide range of functions, including signal transduction, cell proliferation, transcription, apoptosis, neuronal differentiation, cell migration, regulation of ion channels and cell survival (59–62). They are particularly abundant in the brain, where they help with neuronal migration in the cerebral cortex and hippocampus (63). YWHAZ is involved in dopamine production and has been associated with behavioral changes in patients with schizophrenia and cognitive impairment in patients with bipolar disorder. Low expression of YWHAZ can lead to a thicker cortex (62). On the other hand, YWHAG is involved in protein trafficking and neuronal migration, and its upregulation has been associated with delayed neuronal migration during cerebral cortex development (63, 64). Both YWHAZ and YWHAG were differentially expressed in the DLPFC of suicide cases in Kékesi´s study, with opposite effects observed between our samples and hers. Due to the heterogeneity of suicide and associated comorbidities, it is possible that these results differ due to the specificities of the samples.

Alpha-internexin (INA) is a critical component of the cytoskeletal protein network and an essential component of neurofilaments, which are the most abundant protein in the human brain. CNS neurofilaments are heteropolymers composed of INA and low, medium and high molecular weight subunits of the neurofilament protein (65). These subunits play a critical role in synapse formation. Alterations in the expression of alpha-internexin have been associated with various neurological disorders such as schizophrenia, bipolar disorder and Alzheimer’s disease. In Kékesi´s analysis, INA was found to be a differentially expressed protein, with the opposite effect to ours.

It is noteworthy that both the information obtained here and previously suggests that common pathways of neurological development and oxidative stress response are present in the brains of individuals who have died by suicide in all samples studied. It is also interesting to note that all of the information is related to the prefrontal cortex

**Figure 3:**
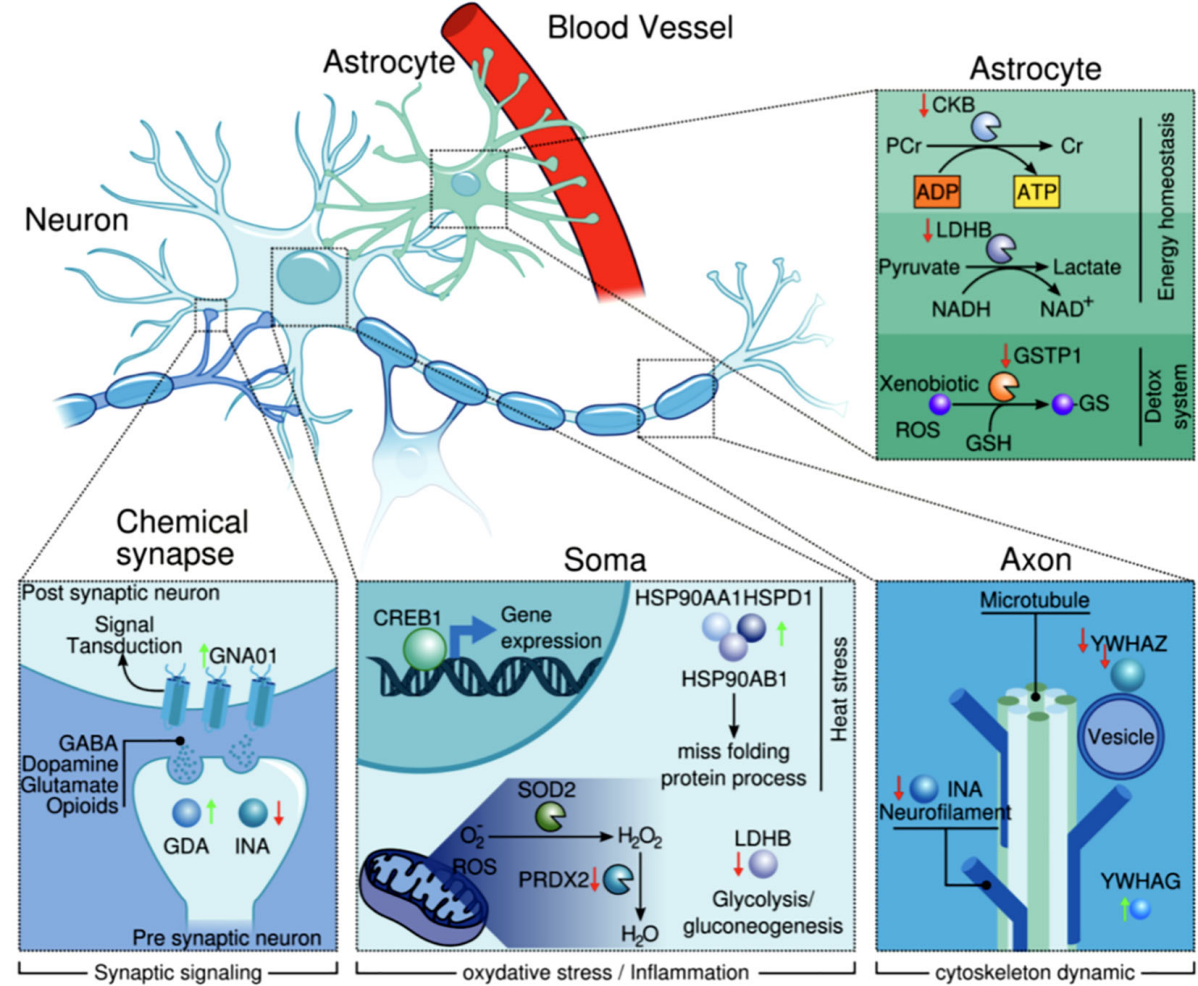
Cellular and subcellular localization of the proteins (according to The Human Protein Atlas). It illustrates how these proteins are interacting and governing relevant biological processes.

Our DEPs were grouped according to their corresponding cellular localization in the Human Protein Atlas, and we have attempted to integrate our results in **Figure 3**. The proteins we analyzed are known to be involved in heat stress, inflammation and oxidative stress responses.

The upregulation of GNAO1 and downregulation of INA and YWHAZ in samples from suicide cases suggest that neurotransmission flux and neurofilament dynamics may be impaired. In addition, GSTP1 and PRDX2 were downregulated, while HSDP1 was upregulated, suggesting oxidative stress.

The upregulation of HSP90 proteins in samples from suicide cases could be due to oxidative stress, while the downregulation of CKB and LDHB negatively affects glycolysis, ATP production and cAMP response. Furthermore, these changes in protein expression in the brain tissue of suicides may guide future studies as they are involved in the response to oxidative stress and in molecular mechanisms related to neurodevelopment. The apparent dysfunction of these proteins in the DLPFC could be linked to suicide.

Our study was able to utilize densitometric data from DEPs to train ML models predicting whether a sample was from individuals who died by suicide or not. The results showed that there was sufficient discrimination between cases and controls. However, to ensure the robustness of this model, it is important to apply it to a larger data set. This step will be carried out as part of the future direction of our study. The ultimate goal is to apply this model to peripheral samples instead of central tissue. The biggest challenge we currently face is to adjust these values to account for the less average densitometric data of dilute samples such as blood, which contains lower amounts of proteins compared to brain tissue.

This conclusion is particularly important if you plan to use these biomarkers for predicting individuals at risk of suicide. Niculescu and cols analyzed the serum proteome (the amount of proteins present in the blood) of individuals with a history of suicide attempts compared to healthy controls (25). The researchers identified several proteins that were differentially expressed in the suicide attempters group, including proteins involved in inflammation and cell adhesion. They were also able to identify subtypes of suicide phenotypes.

It is important to recognize the limitations of this study. The sample size was relatively small, and obtaining human brain tissue samples is a difficult process that requires careful planning and ethical considerations. Despite the limited number of subjects, the study aimed to create a well-characterized cohort of suicide and control subjects consisting of males only, with no statistically significant difference in mean age or PMI. However, it is important to point out that whole tissue corresponding to BA9 of the DLPFC was used for the analysis, which could potentially be a confounding factor as no single cell study was conducted. Furthermore, as MDD was the most common diagnosis in the case group, it is important to verify whether the results are solely related to suicide or whether they are an inherent effect of the psychiatric illness. Although the PCA and heatmap results were not conducted, immunovalidation was performed within another well-characterized cohort from the same source, but only for PRDX2 and INA. Therefore, a deeper understanding of the biological processes involving the DEPs identified in the suicide cases may significantly contribute to uncovering the mechanisms underlying this tragic outcome. It is strongly recommended that the study group and other researchers in this field conduct further investigations and analyses to integrate the available information for a more comprehensive understanding.

Furthermore, it is important to integrate the biologically relevant results with the available social and psychological information on suicide risk factors in order to improve their potential use. Ultimately, this knowledge can be used to develop effective strategies to prevent the loss of precious lives worldwide.

## Limitations of this study

Training a machine learning model on a small dataset presents several challenges that can significantly affect the model’s performance and generalizability. Some primary issues are overfitting and underrepresentation of the data distribution. Therefore, it is necessary to test the machine learning models used in this study using a larger dataset.

## Data Availability

The original contributions presented in the study are included in the article/**Supplementary Material**. Further inquiries can be directed to the corresponding author.
