# Supplementary material for: Proteome analysis of the prefrontal cortex and the application of machine learning models for the identification of potential biomarkers related to suicide
Source: Front Psychiatry. 2025 Feb 20;15:1429953. doi: 10.3389/fpsyt.2024.1429953 (PMC11882514; doi:10.3389/fpsyt.2024.1429953)

SUPPLEMENTARY INFORMATION

Table S1. Quantitation of protein. Protein concentration was obtained from 0.5 g of tissue from DLPFC and was dissolved in 1.5 mL of 50 mM TRIS (p.H. 7.4) buffer.

Sample	Average concentration (μg/ μL)	Total amount (μg)
Control 1	2.73	546
Control 2	4.28	856
Control 3	6.66	1332
Control 4	6.41	1282
Case 1	6.03	1206
Case 2	8.52	1704
Case 3	6.65	1330
Case 4	7.47	1494
Case 5	6.95	1390

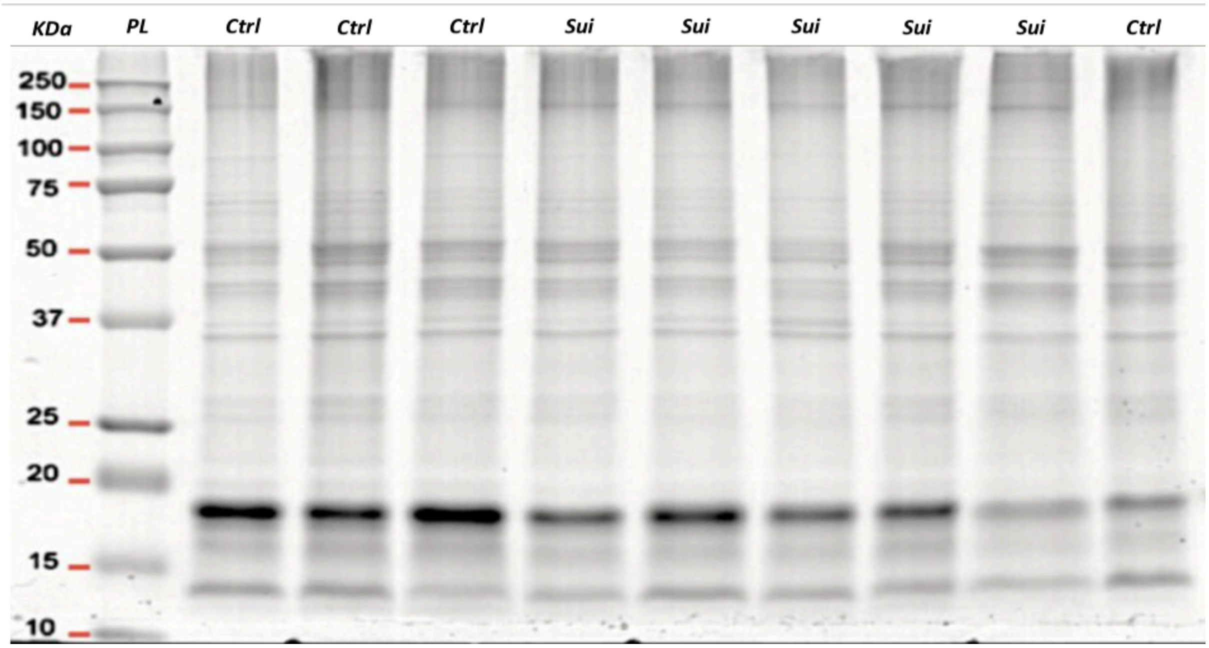

Figure S1: Protein profile by one-dimensional gel electrophoresis. Resolution of proteins based on their molecular weight, from 250 KDa to 10 KDa (Kilodaltons). PL: Protein ladder. Ctrl: Control samples. Sui: samples from suicide.

Table S2. Set of diseases associated with the differentially expressed proteins. Created with MetaCore

#	Disease	p Value	Genes from active data
1	Schizophrenia	1.375×10^{-11}	HSP90AA1, YWHAG, GNAO1, HSPD1, CKB, PRDX2, YWHAZ, GSTP1, INA
2	Schizophrenia spectrum and other psychotic disorders	2.192×10^{-11}	HSP90AA1, YWHAG, GNAO1, HSPD1, CKB, PRDX2, YWHAZ, GSTP1, INA
3	Dementia	5.245×10^{-11}	HSP90AA1, YWHAG, GNAO1, HSP90AB1, PRDX2, HSPD1, CKB, YWHAZ, LDHB, GSTP1, INA
4	Neurocognitive disorders	5.907×10^{-11}	HSP90AA1, YWHAG, GNAO1, HSP90AB1, PRDX2, HSPD1, CKB, YWHAZ, LDHB, GSTP1, INA

5	Alzheimer disease	$7.454x10^{-11}$	HSP90AA1, YWHAG, GNAO1, HSP90AB1, HSPD1, CKB, PRDX2, YWHAZ, GSTP1, INA
6	Tauopathies	$8.322x10^{-11}$	HSP90AA1, YWHAG, GNAO1, HSP90AB1, HSPD1, CKB, PRDX2, YWHAZ, GSTP1, INA
7	Neurodegenerative diseases	$5.473x10^{-10}$	HSP90AA1, YWHAG, GNAO1, HSP90AB1, CKB, PRDX2, HSPD1, YWHAZ, LDHB, GSTP1, INA
8	Brain diseases	$1.445x10^{-9}$	HSP90AA1, YWHAG, GNAO1, HSP90AB1, PRDX2, CKB, HSPD1, YWHAZ, GDA, LDHB, GSTP1, INA
9	Central nervous system diseases	$3.758x10^{-9}$	HSP90AA1, YWHAG, GNAO1, HSP90AB1, HSPD1, CKB, PRDX2, YWHAZ, GDA, LDHB, GSTP1, INA
10	Dyskinesias	$1.395x10^{-8}$	HSDP1, CKB, PRDX2, YWHAZ, LDHB, GSTP1

Figure S2. Immunoblot of the detection of PRDX2 in protein extracts of DLPC of controls (C) and from individual who died by suicide (S)

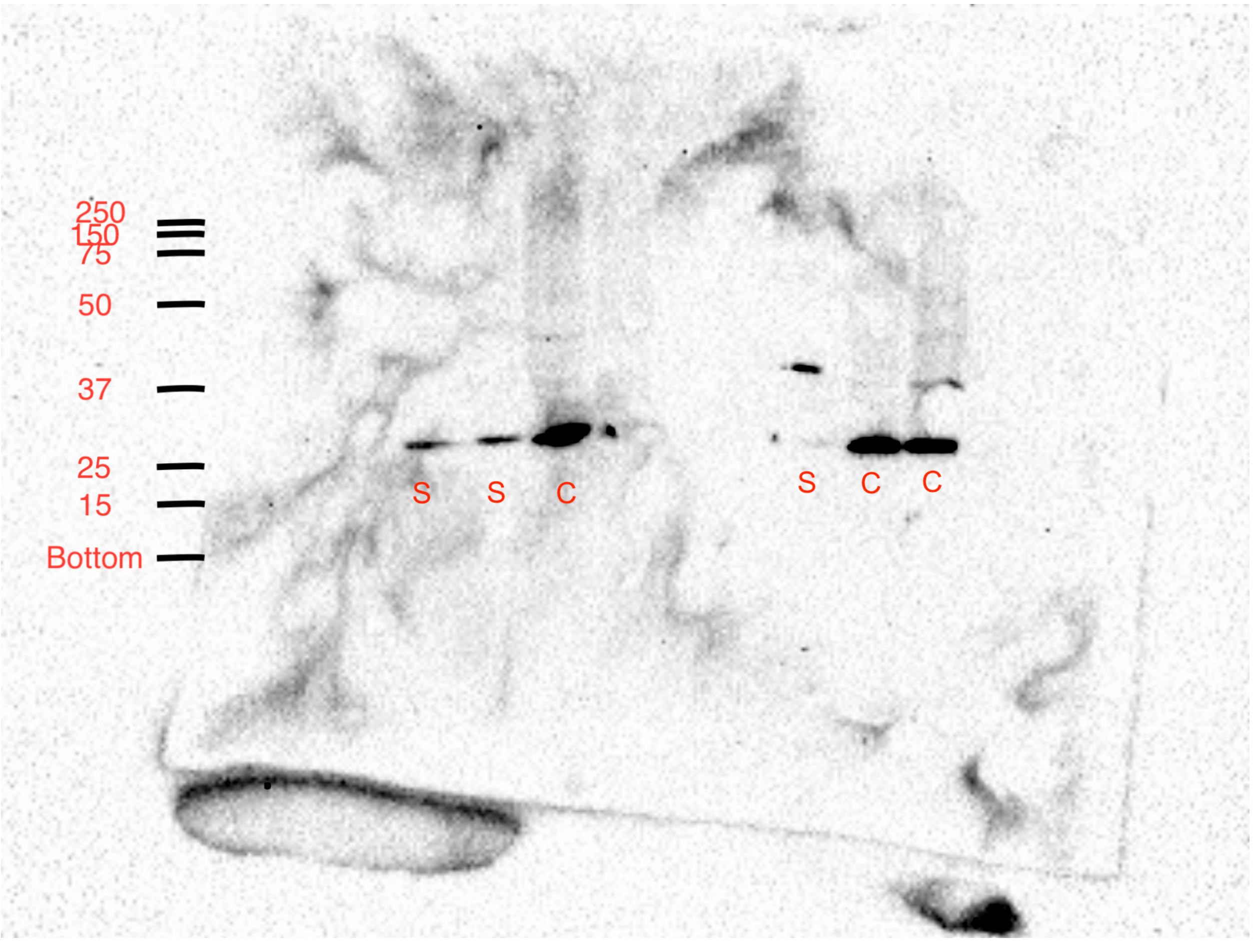

Figure S3. Immunoblot of the detection of INA in protein extracts of DLPC of controls (C) and from individual who died by suicide (S)

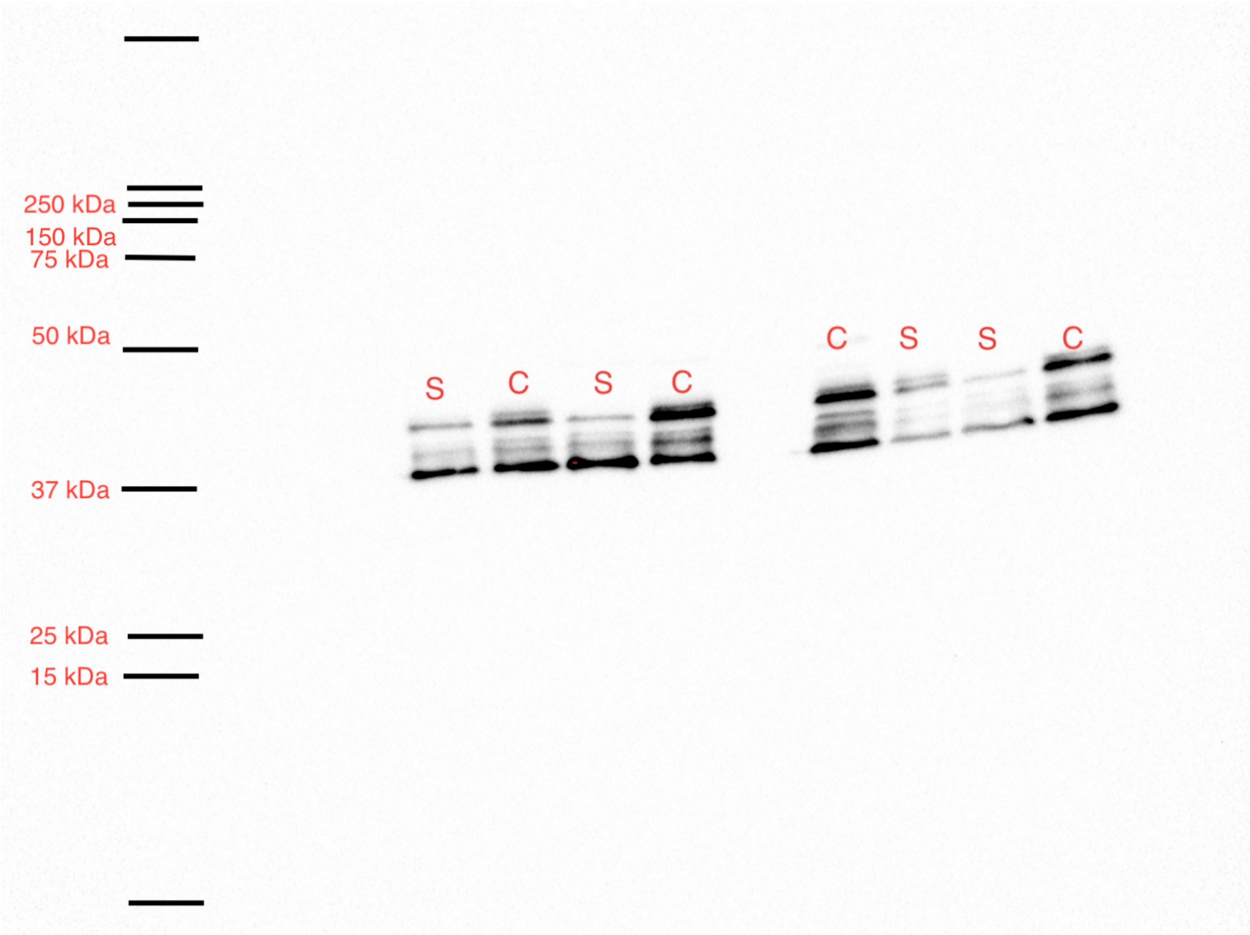

Supplement: Supplementary file 1 [file DataSheet1.pdf]
